# Perception of Cigarette Graphic Health Warnings and Its Impact on Smoking Behavior: A Cross-Sectional Study among Current Smokers of Western Part of Nepal

**DOI:** 10.1155/2022/5787856

**Published:** 2022-09-15

**Authors:** Santosh Shrestha, Santosh Pokhrel, Suraj Subedi, Hemraj Paudel, Rup Chandra Viswakarma, Diptee Poudel, Shiva Lal Bhattarai, Puja Bhandari, Sandip Kuikel

**Affiliations:** ^1^Central Department of Public Administration, Tribhuvan University, Kathmandu, Nepal; ^2^Karnali Academy of Health Sciences, Jumla, Nepal; ^3^Municipal Level Hospital, Tanahun, Nepal; ^4^Pokhara Academy of Health Sciences, Pokhara, Nepal; ^5^Maharajgunj Medical Campus, Tribhuvan University Institute of Medicine, Kathmandu, Nepal

## Abstract

Globally, tobacco use (smoked, secondhand, and chewing) accounted for 8.71 million deaths, which is 15.4% of all deaths in 2019. Tobacco was ranked first among males and sixth among female in terms of level 2 risk factor for attributable deaths globally. The objective of this study was to identify the perception of cigarette graphic health warnings and their impact on smoking behavior in Nepal. A cross-sectional study using purposive sampling technique was done. Out of 169 respondents, 79.9% were male, 49.1% were illiterate, and 37.9% were above 60 years of age. Eighty-four percent had initiated smoking before the age of 20, and 39.6% had smoked cigarettes for more than 40 years. All the respondents had noticed the graphic health warning on cigarette packages. The majority (80.5%) of the respondents reported that the warning informs about specific health consequences of smoking, and the percentage of respondents believing that warning motivates smokers to quit smoking, encourages smokers to reduce the number of cigarettes smoked per day, and deters potential smokers from starting to smoke was 40.2%, 33.1%, and 30.8%, respectively. More than half of the respondents (50.9%) attempted to quit smoking because of the warning. The implementation of graphic health warnings had favorable perception from majority of smokers and positive impact on smoking behavior of the respondents. Further large-scale research on impact on smoking behavior through repeated cross-sectional studies can be future research priority.

## 1. Introduction

Globally, tobacco use (smoked, secondhand, and chewing) accounted for 8.71 million deaths, which is 15.4% of all deaths in 2019. Tobacco was ranked first among males and sixth among female in terms of level 2 risk factor for attributable deaths globally [1].

Tobacco claims 1.6 million lives in the WHO South-East Asia Region (SEAR) alone, which is also among the largest producers and consumers of tobacco products [2]. There is no risk-free level of exposure to tobacco smoke. Tobacco smoke causes adverse health outcomes, particularly cancer and cardiovascular and pulmonary diseases [3]. Persistent cigarette smoking kills about half of its users [4]. Article 11 of the WHO Framework Convention on Tobacco Control (FCTC) requires nations to take effective measures to ensure that tobacco product packaging contains effective health warnings and messages [5]. Similarly, Article 11 Guidelines adopted at the Third Conference of the Parties in November 2008 have put the spotlight on the inclusion of pictures on tobacco package health warnings [6].

The Government of Nepal signed FCTC convention on 3rd December 2003 and House of Representative ratified it on 7th November 2006. The Government of Nepal banned advertisements on hoardings by an Executive Order of March 2010 and also banned advertisements of tobacco products in any media through the Supreme Court verdict of 2006 and 2009. On May 31, 2011, the Government of Nepal passed tobacco packaging regulations which have included requirements for health warnings that cover 75% of both the front and back of the package. The warnings were implemented in April 2014, after a legal challenge with the tobacco industry. Three different warnings were issued for cigarettes, bidis, and smokeless tobacco products such as surti or khaini. Warning text must be in Nepali. Descriptive (qualitative) emission and constituent messages are required on both side panels. In October 2014, the government amended the regulations, increasing the coverage area of the warnings to 90%. The larger warnings were required on packages by May 15, 2015 [7].

According to Nepal STEP Survey 2019, the prevalence of tobacco use among men and women aged 15-69 years was 28.9% (48.3% men and 11.3% women). Out of which, 28% of men and 7.5% of women smoke tobacco while 33.3% of men and 4.9% of women use smokeless tobacco [8]. According to Nepal Demographic and Health Survey, 2016, twenty-seven percent of men use any type of tobacco, as compared with 6% of women [9]. The major forms of tobacco use in Nepal can be divided into smoking tobacco products and smokeless tobacco products. The smoking forms are cigarette, bidi, hookah, sulfa, and chillum or kankad. The smokeless tobacco products include surti leaves, khaini, gutkha, and paan with tobacco ingredients. Tobacco use did not change much between 2013 and 2019 either for total prevalence (30.7% vs. 28.9%) or among men (48.1% vs. 48.3%) or women (14.6% vs. 11.6%). There was similar trend either for those who smoked tobacco, men (27% vs. 28%) and women (10.3% vs. 7.5%), or those who use smokeless tobacco, men (31.4% vs. 33.3%) and women (4.8% vs. 4.9%) [8]. Every year 27,137 people are killed by tobacco-related disease in Nepal which is 14.9% of all deaths of the country. Most of the tobacco caused deaths were due to cardiovascular diseases (CVD) (53%) followed by chronic respiratory diseases (CRD) (21%) and cancers (8%) [10].

Health warning labels are regarded as one of most prominent and cost-effective communication. In many countries, more cigarette smokers report getting information about the health risks of smoking from warning labels than any other source except television [11, 12]. A Toolkit was created to serve as a resource to support implementation of Article 11. It includes a review of evidence, as well as recommendations for designing health warnings on packages. Overall, the Toolkit is intended to simplify the process of developing effective labelling policies and to provide concrete resources for regulators, researchers, and tobacco control advocates [13]. Several published studies, mostly conducted in high-income countries, have confirmed the superiority of graphic health warnings compared to text-based warnings in informing the public about the risks of smoking and stimulating interest in quitting smoking [14–16]. Risk perception has been found to be affected by socioeconomic factors, among which are education and income [17]. According to the Nepal STEP Survey 2019, 75.7% of adults noticed the health warnings on tobacco packages. Among the current users who noticed these health warnings, 44.8% thought of quitting because of the large health warnings [8]. A cross-sectional study done was conducted among 2250 participants in 9 cities between September 2014 and March 2015 in Nepal which showed that participants believed that pictorial health warnings would be effective in motivating smokers to quit (80.2%) and in convincing youth not to start smoking (86.8%), and 58% of the current smokers intended to quit smoking and reduced their daily intake of cigarettes from 11 to 5 on average [18].

There have been few studies in Nepal which has studied the perception of graphic health warnings on smokers and the impact of such pictorial warnings on smoking behavior. The finding of this study will provide data regarding perception of cigarette graphic health warnings and its impact on smoking behavior among residents of Kushma municipality, western part of Nepal. This type of study will be of vital importance to anti-smoking policies and measures, and it will also reinforce the concerned authorities on strong implementation of laws for graphical health warnings.

## 2. Methods

### 2.1. Study Area and Period

The study was carried out in ward number 6 and 7 of Kushma municipality of Parbat district of Nepal from October 12 to November 4, 2021. Parbat district is located in the western region of Nepal. It is one of eleven districts of province 4 or Gandaki province. Parbat district constitutes two municipalities (nagarpalika) and five rural municipalities (gaunpalika). Kushma municipality is one of the two municipalities in the Parbat district, and it has fourteen wards. According to the National Population Census 2021, the district has a total population of 132,703. According to the Nepal Demographic Health Survey 2016, among the 15 to 49 year age group, 6.2% of women and 24.6% of men from Gandaki province smoked cigarette. Since research funding was not available for the study, two wards (ward nos. 6 and 7) of Kushma municipality were selected purposively with consideration of limited time and resources.

### 2.2. Study Design, Study Population, and Exclusion Criteria

A descriptive cross-sectional community-based study was carried out among 169 current smokers in ward nos. 6 and 7 of Kushma municipality of Parbat district of Nepal. The population of interest for the study was current smokers. A current smoker was defined as a person who smoked cigarettes at least once in the past 30 days. Individuals were approached and asked if they had smoked cigarettes at least once in the past 30 days or not. Those who met the mentioned criteria and agreed to participate were included in the study. Individuals who had not smoked cigarettes in the past 30 days were not included in the study.

### 2.3. Data Collection Procedures, Validity, and Reliability

Using a pretested semi-structured interview schedule, a face-to-face interview was carried out for collecting the data. Data collection tool was adapted from earlier literatures [19, 20].

Since the sampling frame or the listing of members of the population under study, i.e., list of current smokers of study area, was not available, a non-probability sampling technique had to be used. Purposive sampling technique was used as the researcher had to identify the sample based on the pre-determined criteria. The sampling for proportionality was not the primary concern, and there were a limited number of people that had the characteristics under study.

A principal investigator was self-involved in collecting the data. At first, the ward administrative office in each ward was located; then, data collection was done by visiting door to door in every household and retail shops which were located in the same side of the ward office. It took ten to fifteen minutes to complete data collection from each respondent.

Out of the 227 current smokers approached for the study, 55 (24.2%) refused to participate in the study, and 3 eligible smokers were unable to complete the study because of cognitive difficulties and were not included in the study, leaving a final sample size of 169.

The interview schedule, which was in English language, was translated into Nepali and again retranslated into English language to find misinterpretation, and then, necessary correction was made.

To test the reliability of interview schedule and assess its validity, pretesting was done in 10% of sample size in ward no. 5 of Kushma municipality and necessary improvements were made in the questionnaire based on the feedback from respondents as well as experts in the same field. Data coding was done on the same day of data collection to simplify the process of data entry. Data compiling, checking, and editing were done to maintain consistency and to find out omissions or repetitions in the same day of data collection.

### 2.4. Study Population Size

Study population size was calculated using finite population correction formula as given below.

(1) $$\begin{array}{c} \text{Study population size }\left(n\right)\text{: }\;N=\frac{z^{2}pq}{d^{2}}, \end{array}$$

where *z* is the standard normal deviation and 1.96 for 95% confidence interval.

A descriptive cross-sectional study “Impact of tobacco health warnings on smokers in Pakistan” conducted by Muhammad Ahsan et al. in Karachi, Pakistan, between July and October 2014 on 1500 male cigarettes smokers of more than 20 years of age revealed that 88.7% participants noticed warning on cigarette packets. If we draw a *p* value from this study, we have

(2) $$\begin{array}{c} p=0.887,\\ q=0.113,\\ d=\text{allowable error}=0.05. \end{array}$$

Now, the required sample size on calculation is 154.

Nonresponse set as 10% is 15.

Hence, the total study population size was 169.

### 2.5. Data Processing and Analysis

Data entry and analysis were done by using SPSS version 20.0. Descriptive analysis was performed as per the study variables to calculate frequency, percentage, mean, and standard deviation. Bivariate analysis was done applying the Pearson chi-square test to find out association between sociodemographic variables and change in smoking behavior due to graphic health warnings.

### 2.6. Ethical Considerations

Ethical approval for the study was obtained from the Institutional Review Committee of the Institute of Medicine. All information collected was utilized only for study purpose. Confidentiality of the respondent was maintained during data collection and analysis of the study. The study was explained to participants, and verbal consent was taken for data collection. Research codes were used instead of the participant's name to ensure anonymity. The participation was completely voluntary. Participant's right to refuse to participate in the study was respected. Participants were free to quit at any time they like.

### 2.7. Operational Definitions

Awareness of graphic health warnings refers to the state of being aware of graphic health warnings on cigarette packages and any regulation regarding the provision to use graphic health warnings on cigarette packages.

Perception of graphic health warning refers to the way in which graphic health warning on cigarette packages is understood or interpreted.

Graphic health warnings refer to the photographic image printed on the tobacco product package which accurately depicts the hazards of tobacco use and is accompanied by textual warnings related to the picture [21].

A current smoker is defined as a person who smoked cigarettes at least once in the past 30 days. A light smoker is defined as those who smoke ten or less cigarettes per day and heavy smokers as those who smoke more than 10 cigarettes per day on days smoked.

### 2.8. Limitations

Since the study was done in selected wards of a municipality, it may not represent the scenario of a whole district. But the literacy rate, socioeconomic status, cultural and religious diversity, etc. of the interviewee are comparable to those of the other wards of the district and general Nepalese population. Therefore, study results are somehow generalizable.

As the study is cross-sectional, it cannot draw causal relationship on the basis of study results. Similarly, potential confounders were not studied so result could not be adjusted for confounders. Since there is no randomization in purposive sampling, the members of the population under study do not have equal chance of selection. It results in a bias in information, and sample collected may not adequately be representative of the population under study. Similarly, the study did not include past smokers who quit smoking after seeing graphic health warnings on cigarette packages.

## 3. Results

A large number of respondents were above 60 years (37.9%), followed by the 20.1% of age group 20 to 29 years, 16.6% of age group 50 to 59 years, 10.7% of age group 30 to 39 years, and 5.9% of age group 40 to 49 years. The mean age was 46.95 years. Majority of the respondents were male (79.9%), Hindu (96.4%), and illiterate (49.1%). Almost half of the respondents (49.7%) were from the Dalit ethnic group (49.7%), followed by 25.4% which were Janajati and the remaining 24.9% from the upper caste group. Most of the respondents, i.e., 84%, had initiated smoking before the age of 20 years, majority of the respondents (82.8%) were light smokers (smoking 1-10 cigarette sticks per day), a total of 67.5% of the respondents reported that they spend less than thousand rupees on smoking every month, and when asked about how long it has been since they started smoking, 39.6% reported more than 40 years (Table 1).

Nearly all (99.4%) of the respondents agreed that graphic health warning on cigarette package is noticeable, followed by the warning is informative (93.5%) and warning is believable (80.5%). More than half of the respondents (51%) felt that it is necessary to keep graphic health warning on packages of cigarette, and 66.3% of respondents had thought about what graphic health warning has to say. Further, 36.1% of the respondents believed that graphic health warning makes smokers more likely to quit smoking. Most of the respondents (88.2%) agreed that the warnings have increased awareness on health consequences of smoking, 89.3% agreed the warnings depict the health consequences of smoking, and 85.8% reported the warnings made them think about the health consequences of smoking. Majority of the respondents (63.3%) reported that they had talked about the warnings with other smokers or nonsmokers. Similarly, 93.5% of the respondents thought the warnings made the cigarette packages less attractive and 91.7% of them thought the warnings made smoking less attractive. All the respondents, i.e., 100%, had noticed the graphic health warning on cigarette package. Majority of respondents (92.9%) had not heard of any regulation regarding provision of graphic health warning. Majority of the respondents (80.5%) reported that the warning informs about specific health consequences of smoking, warning motivates smokers to quit smoking (40.2%), warning encourages to reduce no. of sticks smoked per day (33.1%), warning deters potential smokers from starting to smoke (30.8%), and 7.1% reported that warning does not have any effect (Table 2).

There was no significant association between attempt to quit smoking due to graphic health warnings and different sociodemographic variables (*p* value ≥ 0.05), and there is no significant association between attempt to quit smoking due to graphic health warnings and different sociodemographic variables and smoking history of the respondents (*p* value ≥ 0.05). Had thought about what graphic health warning has to say was significant with attempt to quit smoking due to graphic health warning; however, talking about graphic health warning with other smokers or nonsmokers is not significantly associated with attempt to quit due to graphic health warning (Table 3).

There was no significant association between decrease in number of cigarettes smoked per day due to graphic health warnings and different sociodemographic variables. Decrease in no. of cigarette sticks smoked per day was statistically significant with duration of smoking (*p* = 0.036), no. of sticks smoked per day (*p* = 0), and expenditure on smoking per month (*p* = 0). There is no significant association found with sociodemographic variable, smoking history, and decrease in no. of cigarette sticks smoked per day. There is no significant association between thought about and talked about graphic health warnings and plan to quit smoking due to graphic health warnings (Table 3).

There was significant association between plan to quit smoking within 6 months due to graphic health warnings and age of the respondents (*p* = 0) and education (*p* = 0). There is no significant association found with other sociodemographic variables and plan to quit smoking within 6 months due to graphic health warnings. There was statistical significance between plan to quit smoking within 6 months due to graphic health warnings and duration of smoking (*p* = 0.001). There is no significant association found with other smoking histories of the respondents and plan to quit smoking within 6 months due to graphic health warnings (Table 3).

## 4. Discussion

Our study showed that most of the respondents, i.e., 84%, had initiated smoking before the age of 20 years. This is similar to the study conducted in the Rupandehi district of Nepal by Mishra et al. with 86% of the respondents initiating smoking before 18 years of age. Most of the respondents had parents and grandparents who were smokers, and their first exposure to cigarette smoking was when their parents and grandparents asked them to light a cigarette [19]. A study conducted among adolescents by Paudel et al. in Pokhara, Nepal, found that adolescents from families with at least one member using tobacco were 1.79 times more likely to use tobacco compared to those with no members using tobacco. When adolescents are exposed to the tobacco use habit of family members, they are more likely to perceive tobacco use as a positive and acceptable behavior [22].

All the respondents had noticed the graphic health warning on cigarette packets. The study by Mishra et al. showed that 79.3% of the respondents had heard of graphic health warnings. This may be because the graphic health warnings were implemented three months before the study. Now, it has been more than seven years since the first implementation of graphic health warnings on tobacco products. At present, almost all smokers and nonsmokers are aware of graphic health warnings on tobacco products [19].

The Global Adult Tobacco Survey (GATS) is a nationally representative household survey conducted among persons aged ≥15 years. GATS was conducted once in 14 countries (Bangladesh, Brazil, China, Egypt, India, Mexico, Philippines, Poland, Russia, Thailand, Turkey, Ukraine, Uruguay, and Vietnam). In all countries except India (78.4%) and Mexico (83.5%), more than 90% of men reported noticing a health warning on a cigarette package. Among women, the percentage who noticed warnings was 75% or more in all countries except China (60.1%) and India (18.9%) and more than 90% in seven countries. In Bangladesh and Egypt, very few women reported current smoking to calculate this percentage [23]. A cross-country comparison of health warnings across 19 countries (Australia, Bangladesh Brazil, Canada China, France, Germany, Ireland, Malaysia, Mauritius, Mexico, Netherlands, New Zealand, Scotland, South Korea, Thailand, United Kingdom, United States, and Uruguay) by the International Tobacco Control Policy Evaluation Project (the ITC Project) revealed that the percentage of smokers who noticed the health warnings on cigarette packages “often” or “very often” was the highest in Mauritius (81.9%) and the lowest in Netherlands (16.4%). This may be due to the fact that Netherlands had text-only warnings at the time of survey in 2011 covering only 30% of the front and 40% of the back of the cigarette packet, whereas Mauritius had pictorial warnings covering 60% of the front and 70% of the back of the cigarette packet [24].

Our study findings showed that when asked about the effect of keeping graphic health warnings on cigarette packages majority of the respondents (80.5%) reported that the warning informs about specific health consequences of smoking, warning motivates smokers to quit smoking (40.2%), warning encourages to reduce no. of sticks smoked per day (33.1%), warning deters potential smokers from starting to smoke (30.8%), and 7.1% reported that warning does not have any effect.

The study by Mishra et al. showed that 32.7% of the respondents reported that graphic health warnings encourage to stop smoking, 30% reported it informs about health hazards of smoking, 15% felt it does not have any effect, and 9.3% reported it decreases the number of sticks of cigarette smoked per day [19]. The difference in finding may be because this study was conducted in a rural area, with high illiteracy (49.1%), unlike the study conducted by Mishra et al. where only 16% of the respondents were illiterate, and the study was conducted in an urban area, i.e., Butwal submetropolitan city [19].

Inquiries regarding perception of graphic health warnings revealed that nearly all (99.4%) of the respondents agreed that graphic health warning on cigarette package is noticeable, followed by the warning is informative (93.5%) and warning is believable (80.5%). More than half of the respondents (51%) felt that it is necessary to keep graphic health warning on packages of cigarette, and 66.3% of the respondents had thought about what graphic health warning has to say. Further, 36.1% of the respondents believed that graphic health warning makes smokers more likely to quit smoking attractive. The study by Mishra et al. showed that about 73% of the respondents felt the warning is noticeable, followed by the warning is informative (72%), the warning is believable (68%), and necessary to keep the warning on packages of cigarette (81.3%) [19]. A similar study conducted in Canada by Hammond et al. revealed that 74.5% of the respondents thought about what the warning on the outside of the packages has to say [25]. Abdolahinia et al. concluded that a total of 62% of smokers supported the placement of pictures and 8% stated that seeing the pictorial warning motivated them to quit smoking [26]. The cross-country comparison of health warnings across 19 countries of the ITC Project revealed that the percentage of smokers who said warning labels made them think about the health risks of smoking “a lot” by country was the highest in Brazil (44.5%) and the lowest in the Netherlands (1.4%) with text-only health warnings. Similarly, the percentage of smokers who made them “a lot” more likely to quit smoking was the highest in Thailand (36.7%) and the lowest in Netherlands (1%) with text-only warning labels [24].

Majority of the respondents (63.3%) reported that they had talked about the warnings with other smokers or nonsmokers. A similar study conducted in Canada by Hammond et al. showed that 81.1% of the respondents had ever talked about the graphic health warnings with other smokers or nonsmokers [25]. Most of the respondents (88.2%) agreed that the warnings have increased awareness on health consequences of smoking, 89.3% agreed the warnings depict the health consequences of smoking, and 85.8% reported the warnings made them think about the health consequences of smoking. Similarly, 93.5% of the respondents thought the warnings made the cigarette packages less attractive and 91.7% of them thought the warnings made smoking less attractive. ITC research across four countries also showed that text-only warnings were associated with lower levels of awareness about the health risks of smoking than prominent, graphic warnings [27].

Our study finding revealed that when asked about what their behavior was since they started to smoke from packages with graphic health warnings, respondents indicated the following actions: stub-out cigarette before smoked completely (7.7%), forego a cigarette (2.4%), smoke less around other people (20.1%), avoid looking at the warning labels (50.9%), think about quitting smoking (53.8%), and feel more vulnerable after seeing the pack (58.6%). Only 11.8% of the respondents reported that they had not changed their smoking behavior due to graphic health warnings.

The results from GATS which was conducted among persons aged ≥15 years in 14 countries revealed that, among smokers who noticed a package warning, the percentage thinking about quitting because of the warning was >50% in six GATS countries (Bangladesh, Brazil, India, Thailand, Ukraine, and Vietnam) and >25% for men and women in all countries except one (Poland). Older male smokers were less likely to think about quitting in India and Uruguay; no other age group differences were noted [28].

The cross-country comparison of health warnings across 19 countries of the ITC Project revealed that the percentage of smokers who made an effort to avoid warning labels was the highest in Thailand (55.5%) and the lowest in Germany (2.3%). Similarly, the percentage of smokers who gave up a cigarette at least once because of the warning labels was the highest in Thailand (55.5%) and the lowest in Germany (6.7%). Thailand had graphic health warnings occupying 55% of the front and back of the packet, and Germany had text-only health warnings occupying 30% of the front and 40% of the back of the packet [22]. The result of the impact of graphic health warnings was similar to previously published studies. The Flash Euro barometer on Tobacco (Flash No 253) survey of 26,500 citizens aged 15 years and over showed that three out of ten (31%) citizens think that health warnings on tobacco packs are effective in informing them about the health effects of tobacco. Three out of ten (31%) nonsmokers perceive health warnings as being effective in preventing them from smoking, and a fifth of smokers perceive them as being effective in persuading them to smoke less or to try to quit smoking (22% and 19%, respectively). Younger respondents, the less-educated respondents, and manual workers across all groups (those who have never smoked, former smokers, and current smokers) appear to be slightly more likely to perceive health warnings on tobacco packs as being effective [29].

Current smokers who had thought about what graphic health warnings have to say were significantly associated with attempt to quit smoking and decrease in number of cigarette sticks smoked per day. This finding supports the study conducted in Canada by Hammond et al. which showed smokers who had read, thought about, and discussed the warning labels were more likely to either attempt to quit or reduce their smoking [25].

Through bivariate analysis, we found that the age of respondents was significantly associated with a decrease in the number of cigarette sticks smoked per day due to graphic health warnings. It reveals that current smokers of age 40-49 years are most likely and smokers above 60 years of age are least likely to decrease the no. of cigarette sticks smoked per day due to graphic health warnings. The duration of smoking was also significantly associated with decrease in number of cigarettes smoked per day. It showed current smokers who are smoking since teenage and those who were smoking for more than 50 years were least likely to decrease the number of cigarettes smoked per day due to graphic health warnings.

Similarly, a decrease in number of cigarettes smoked per day was significantly associated with number of cigarettes smoked per day and expenditure on smoking per month. It revealed current smoker who smoked less than ten cigarettes per day was likely to decrease number of cigarette sticks smoked per day than that who smoke more than ten cigarettes per day. Similarly, those who expend less than a thousand rupees per month were more likely to decrease in number of cigarettes than those who spend more than thousand rupees per month on smoking. Duration of smoking was associated with plan to quit smoking within six months due to graphic health warnings. Those who were smoking since less than ten years were more likely have a plan to quit smoking due to graphic health warnings.

More than 80% respondents agree that GHWs are informative and believable, but only 51% felt that it is necessary to keep GHWs on cigarette packages. This shows that people do not want to see alarming images before they smoke. The ultimate goal of the GHWs is either to reduce the no. of cigarette sticks smoked per day or to quit altogether. Our study showed only 36.1% smokers believe these warnings make smoker to quit which mean people are theoretically agreeing with the goal of putting GHWs on cigarette packages, but when it comes to behavior change, there is still big no. Talking about other aspect of impact of GHWs, only 11.8% had not changed their smoking behavior after seeing GHWs which means a large proportion of smokers are changing their smoking behavior at least some which is a very good part but they are still smoking. Our study also showed that 65.1% of the respondents would smoke even if the price of cigarettes gets doubled. This particularly shows the possible outcome of increased taxation on cigarette products. People are ready to spend more money on cigarettes. So, increase in taxation of tobacco product is not the only solution to reduce smoking behavior. All dimensions of the measures should be adopted to reduce the global pandemic of cigarette smoking. As the study did not include past smokers, we could not say about how many people actually stopped smoking due to GHWs on cigarette packages.

As our study was conducted in a small population of two wards of Kushma municipality of Parbat district, it may not represent the whole Nepalese adult population. So, our advice would be to pick up adequate sample including current and past smokers from each province and to consider all the potential confounders. Nonpurposive sampling, if possible interventional studies, would obviously be able to draw a fine conclusion regarding ground reality of how GHWs on cigarette packages impact smokers. A similar study can be conducted about smokeless tobacco products like bidi, khaini, and surti.

## 5. Conclusion

The results showed that most of the respondents reported that graphic health warnings inform about specific consequences of smoking; most felt the warnings are informative, believable, have increased awareness of the consequences of smoking, and made them think about the health consequences of smoking. However, less than half of them believed the warnings motivated smokers to quit smoking and felt that the warnings encouraged smokers to reduce no. of cigarette sticks smoked per day and it may deter people from starting to smoke. Similarly, only half of the respondents attempted to quit smoking due to graphic health warnings, and less than half had planned to quit smoking due to graphic health warnings. This difference between high perception but relatively low level of impact on smoking behavior may be due to the addictive nature of tobacco products, deep-rooted social norms of smoking, initiation of smoking at an early age, and because almost half of the respondents were illiterate and from disadvantaged Dalit ethnic group.

Since half of the respondents had attempted to quit smoking, more than a third of the respondents had a plan to quit smoking, and almost two-thirds of respondents thought about graphic health warnings and talked about it with other smokers or nonsmokers, the implementation of graphic health warnings had favorable perception from most smokers and positive impact on smoking behavior of the respondents.

The study showed a favorable perception and positive impact on the smoking behavior of current smokers due to graphic health warnings. So, the implementation of regulations and standards regarding graphic health warnings on tobacco products should be monitored. Strategic activities of the tobacco industry to dampen the effect of tobacco control policies and programs should be adequately addressed. Since the study was done in selected wards of a municipality, it may not be generalized but it opens a potential space for further studies.

## Figures and Tables

**Table 1:** Sociodemographic characteristics and smoking history of the respondents.

Characteristic	Frequency (*n* = 169)	Percent
Age in years		
<20	15	8.9
20-29	34	20.1
30-39	18	10.7
40-49	10	5.9
50-59	28	16.6
≥60	64	37.9
Mean ± SD	46.95 ± 19.46	
Sex		
Male	135	79.9
Female	34	20.1
Ethnicity		
Dalit	84	49.7
Janajati	43	25.4
Upper caste group	42	24.9
Religion		
Hindu	163	96.4
Buddhist	6	3.6
Education		
Illiterate	83	49.1
Primary	42	24.9
Lower secondary	16	9.5
Secondary	21	12.4
Higher secondary	7	4.1
Age of smoking initiation (years)		
<20	142	84
20-30	25	14.8
>30	2	1.2
Mean ± SD	16.72 ± 4.12	
Duration of smoking (years)		
<10	34	20.1
10-19	29	17.2
20-29	19	11.2
30-39	20	11.8
≥40	67	39.6
No. of cigarette sticks smoked per day		
1-10	140	82.8
11-30	29	17.2
Expenditure on smoking per month (in rupees)		
<1000	114	67.5
1000-1999	49	29
≥2000	6	3.6

**Table 2:** Perception of graphic health warning and its impact on smokers.

Characteristics	Frequency (*n* = 169)	Percent
Graphic health warning on cigarette package is noticeable	168	99.4
Graphic health warning on cigarette package is informative	158	93.5
Graphic health warning on cigarette package is believable	136	80.5
Felt that it is necessary to keep graphic health warning on package of cigarette	87	51.5
Gave a thought to what graphic health warning has to say	112	66.3
Warnings make smokers more likely to quit smoking	61	36.1
Warnings have increased awareness on health consequences of smoking	149	88.2
Warnings depict the health consequences of smoking	151	89.3
Warnings make them think about the health consequences of smoking	145	85.8
Ever talked about the warnings with other smokers or nonsmokers	107	63.3
Warnings make cigarette packages less attractive	158	93.5
Warnings make smoking less attractive	155	91.7
*Impacts of keeping graphic health warning on smokers*		
Informs about specific health consequences of smoking	136	80.5
Motivates smokers to quit smoking	68	40.2
Encourage to reduce no. of sticks smoked per day	56	33.1
Deters potential smokers from starting to smoke	52	30.8
Does not have any effect	12	7.1

**Table 3:** Association between thought about and talked about graphic health warnings with attempted to quit, decrease in no. of cigarette sticks smoked per day, and plan to quit smoking within 6 months.

Characteristics		Attempted to quit smoking due to graphic health warnings		*X* ^2^	*p* value	Crude OR	CI (95%)
		Yes (%)	No (%)				
Thought about what graphic health warning has to say	Yes (%)	70 (62.5)	42 (37.5)	17.917	0.000^∗^	4.271	(2.136-8.540)
	No (%)	16 (28.1)	41 (71.9)				
Talked about graphic health warning with other smokers or nonsmokers	Yes (%)	60 (56.1)	47 (3.9)	3.140	0.076	1.768	(0.939-3.328)
	No (%)	26 (41.9)	36 (58.1)				

		Decrease in no. of cigarette sticks smoked per day					
Yes (%)	No (%)						
Thought about what graphic health warning has to say	Yes (%)	78 (69.6)	34 (30.4)	26.449	0.000^∗^	5.879	(2.907-11.889)
	No (%)	16 (28.1)	41 (71.9)				
Talked about graphic health warning with other smokers or nonsmokers	Yes (%)	68 (63.6)	39 (36.4)	4.431	0.006^∗^	2.414	(1.273-4.578)
	No (%)	26 (41.9)	36 (58.1)				

		Plan to quit smoking within 6 months					
Yes (%)	No (%)						
Thought about what graphic health warning has to say	Yes (%)	22 (19.6)	90 (80.4)	0.814	0.367	1.97	(0.621-3.613)
	No (%)	8 (14)	49 (86)				
Talked about graphic health warning with other smokers or nonsmokers	Yes (%)	22 (20.6)	85 (79.4)	1.577	0.209	1.747	(0.726-4.2040)
	No (%)	8 (12.9)	54 (87.1)				

^∗^Statistically significant at *p* < 0.05.

## Data Availability

All the data are available upon reasonable request to the corresponding author.

## Supplementary Materials

Supplementary Materials Supplementary Content 1: supporting tables. Table A: association between attempts to quit smoking and sociodemographic variables. Table B: association between attempt to quit smoking and sociodemographic variables (continued). Table C: association between decrease in no. of sticks and sociodemographic variables. Table D: association between decrease in no. of sticks and sociodemographic variables. Table E: association between plan to quit smoking within 6 months and smoking history. Supplementary Content 2: ethnicity classification. Supplementary Content 3: informed consent form in English. Supplementary Content 4: interview questionnaire in English.
