# Supplementary material for: Perception of Cigarette Graphic Health Warnings and Its Impact on Smoking Behavior: A Cross-Sectional Study among Current Smokers of Western Part of Nepal
Source: J Smok Cessat. 2022 Sep 15;2022:5787856. doi: 10.1155/2022/5787856 (PMC9499817; doi:10.1155/2022/5787856)
Supplement: Supplementary Materials — Supplementary Content 1: supporting tables. Table A: association between attempts to quit smoking and sociodemographic variables. Table B: association between attempt to quit smoking and sociodemographic variables (continued). Table C: association between decrease in no. of sticks and sociodemographic variables. Table D: association between decrease in no. of sticks and sociodemographic variables. Table E: association between plan to quit smoking within 6 months and smoking history. Supplementary Content 2: ethnicity classification. Supplementary Content 3: informed consent form in English. Supplementary Content 4: interview questionnaire in English. [file 5787856.f1.docx]

# Supplementary Content

## Supplementary Content 1: Supporting tables

Table A: Association between attempts to quit smoking and socio-demographic variables

| Characteristics | | Attempt to quit smoking | | X² | P value |
| --- | --- | --- | --- | --- | --- |
|  |  | Yes (%) | No (%) |  |  |
| Age |  |  |  | 6.034 | 0.303 |
|  | < 20 | 10 (66.7) | 5 (33.3) |  |  |
|  | 20 – 29 | 19 (55.9) | 15 (44.1) |  |  |
|  | 30 – 39 | 9 (50) | 9 (50) |  |  |
|  | 40 – 49 | 7 (70) | 3 (30) |  |  |
|  | 50 – 59 | 10 (35.7) | 18 (64.3) |  |  |
|  | ≥ 60 | 31 (48.4) | 33 (51.6) |  |  |
| Sex |  |  |  | 0.250 | 0.617 |
|  | Male | 70 (51.9) | 65 (48.1) |  |  |
|  | Female | 16 (47.1) | 18 (52.9) |  |  |
| Ethnicity |  |  |  | 0.442 | 0.802 |
|  | Dalits | 44 (52.4) | 40 (47.6) |  |  |
|  | Janajatis | 20 (46.5) | 23 (53.5) |  |  |
|  | Upper caste groups | 22 (52.4) | 20 (7.6) |  |  |
| Education |  |  |  | 8.314 | 0.081 |
|  | Illiterate | 38 (45.8) | 45 (54.2) |  |  |
|  | Primary | 20 (47.6) | 22 (52.4) |  |  |
|  | Lower secondary | 9 (56.2) | 7 (43.8) |  |  |
|  | Secondary | 12 (57.1) | 9 (42.9) |  |  |
|  | Higher secondary | 7 (100) | 0 (0) |  |  |
| Occupation |  |  |  | 0.376 | 0.540 |
|  | Clerical,shop-owner, farmer | 65 (49.6) | 66 (50.4) |  |  |
|  | Other | 21 (55.3) | 17 (44.7) |  |  |

Table B: Association between attempt to quit smoking and socio-demographic variables (continued)

| Characteristics | | Attempt to quit smoking | | X² | P value |
| --- | --- | --- | --- | --- | --- |
|  |  | Yes (%) | No (%) |  |  |
| Family type | |  |  | 2.974 | 0.085 |
|  | Nuclear | 17 (39.5) | 26 (60.5) |  |  |
|  | Joint | 69 (54.8) | 57 (45.2) |  |  |
| Monthly income of the family | |  |  | 16.295 | 0.006 |
|  | 2301 - 6850 | 6 (27.3) | 16 (72.7) |  |  |
|  | 6851 - 11450 | 22 (40) | 33 (60) |  |  |
|  | 11451 - 17150 | 14 (56) | 11 (44) |  |  |
|  | 17151 - 22850 | 15 (55.6) | 12 (44.4) |  |  |
|  | 22851 - 45750 | 19 (67.9) | 9 (32.1) |  |  |
|  | >45751 | 10 (83.3) | 2 (16.7) |  |  |
| Age of smoking initiation (years) | |  |  | 0.901 | 0.342 |
|  | < 20 | 70 (49.3) | 72 (50.7) |  |  |
|  | 20 – 40 | 16 (59.3) | 11 (40.7) |  |  |
| Duration of smoking (years) | |  |  | 3.602 | 0.608 |
|  | < 10 | 21 (61.8) | 13 (38.2) |  |  |
|  | 10 - 19 | 14 (48.3) | 15 (51.7) |  |  |
|  | 20 – 29 | 10 (52.6) | 9 (47.4) |  |  |
|  | 30 – 39 | 11 (55) | 9 (45) |  |  |
|  | 40 – 49 | 17 (50) | 17 (50) |  |  |
|  | >50 | 13 (39.4) | 20 (60.6) |  |  |
| No. of cigarette sticks smoked per day | | |  | 2.351 | 0.125 |
| 1-10 | | 75 (53.6) | 65 (46.4) |  |  |
| 11-30 | | 11 (37.9) | 18 (62.1) |  |  |
| Expenditure on smoking per month (in NRs.) | | |  | 1.715 | 0.190 |
| < 1000 | | 62 (54.4) | 52 (45.6) |  |  |
| ≥ 1000 | | 24 (43.6) | 31 (56.4) |  |  |

Table C: Association between decrease in no. of sticks and socio-demographic variables

| Characteristics | | Decrease in number of cigarette sticks smoked per day | | X² | P value |
| --- | --- | --- | --- | --- | --- |
|  |  | Yes (%) | No (%) |  |  |
| Age |  |  |  | 14.547 | 0.012* |
|  | < 20 | 7 (46.7) | 8 (53.3) |  |  |
|  | 20 – 29 | 22 (64.7) | 12 (35.3) |  |  |
|  | 30 – 39 | 13 (72.2) | 5 (27.8) |  |  |
|  | 40 – 49 | 9 (90) | 1 (10) |  |  |
|  | 50 – 59 | 17 (60.7) | 11 (39.3) |  |  |
|  | ≥ 60 | 26 (40.6) | 38 (59.4) |  |  |
| Sex |  |  |  | 0.545 | 0.460 |
|  | Male | 77 (57) | 58 (43) |  |  |
|  | Female | 17 (50) | 17 (50) |  |  |
| Ethnicity | |  |  | 4.116 | 0.128 |
|  | Dalits | 52 (61.9 ) | 32 (38.1) |  |  |
|  | Janajatis | 24 (55.8) | 19 (44.2) |  |  |
|  | Upper caste groups | 18 (42.9) | 24 (57.1) |  |  |
| Education | |  |  | 9.496 | 0.050 |
|  | Illiterate | 46 (55.4) | 37 (44.6) |  |  |
|  | Primary | 18 (42.9) | 24 (57.1) |  |  |
|  | Lower secondary | 11 (68.8) | 5 (31.2) |  |  |
|  | Secondary | 12 (57.1) | 9 (42.9) |  |  |
|  | Higher secondary | 7 (100) | 0 (0) |  |  |
| Occupation | |  |  | 1.128 | 0.288 |
|  | Clerical, shop-owner, farmer | 70 (53.4) | 61 (46.6) |  |  |
|  | Other | 24 (63.2) | 14 (36.8) |  |  |

Table D: Association between decrease in no. of sticks and socio-demographic variables

| Characteristics |  | Decrease in no. of cigarette sticks smoked per day | | X² | P value |
| --- | --- | --- | --- | --- | --- |
|  |  | Yes (%) | No (%) |  |  |
| Family type |  |  |  | 0.106 | 0.744 |
|  | Nuclear | 23 (53.5) | 20 (46.5) |  |  |
|  | Joint | 71 (56.3) | 55 (43.7) |  |  |
| Monthly income of the family | |  |  | 6.944 | 0.225 |
|  | 2301 - 6850 | 10 (45.5) | 12 (54.5) |  |  |
|  | 6851 - 11450 | 28 (50.9) | 27 (49.1) |  |  |
|  | 11451 - 17150 | 18 (72) | 7 (28) |  |  |
|  | 17151 - 22850 | 12 (44.4) | 15 (55.6) |  |  |
|  | 22851 - 45750 | 18 (64.3) | 10 (35.7) |  |  |
|  | >45751 | 8 (66.7) | 4 (33.3) |  |  |
| Age of smoking initiation (years) | |  |  |  |  |
|  | < 20 |  |  |  |  |
|  | 20 – 40 |  |  |  |  |
| Duration of smoking (years) | |  |  | 11.948 | 0.036* |
|  | < 10 | 19 (55.9) | 15 (44.1) |  |  |
|  | 10 - 19 | 21 (72.4) | 8 (27.6) |  |  |
|  | 20 – 29 | 14 (73.7) | 5 (26.3) |  |  |
|  | 30 – 39 | 12 (60) | 8 (40) |  |  |
|  | 40 – 49 | 16 (47.1) | 18 (52.9) |  |  |
|  | >50 | 12 (36.4) | 21 (63.6) |  |  |
| No. of cigarette sticks smoked per day | | |  | 38.604 | 0.000* |
|  | 1 - 10 | 93 (66.4) | 47 (33.6) |  |  |
|  | 11 - 30 | 1 (3.4) | 28 (96.6) |  |  |
| Expenditure on smoking per month (in NRs.) | | |  | 14.672 | 0.000* |
|  | < 1000 | 75 (65.8) | 39 (34.2) |  |  |
|  | ≥ 1000 | 19 (34.5) | 36 (65.5) |  |  |

Table E: Association between plan to quit smoking within 6 months and smoking history

| Characteristics |  | | Plan to quit smoking within 6 months | | X² | P value |
| --- | --- | --- | --- | --- | --- | --- |
| Age of smoking initiation (years) | | |  |  | - | 0.548* |
|  | < 20 | | 25 (17.6) | 117 (82.4) |  |  |
|  | 20 – 40 | | 5 (18.5) | 22 (81.5) |  |  |
| No. of cigarette sticks smoked per day | | | |  | 1.315 | 0.251 |
|  | 1 – 10 | | 27 (19.3) | 113 (80.7) |  |  |
|  | 11 – 30 | | 3 (10.3) | 26 (89.7) |  |  |
| Expenditure on smoking per month (in NRs.) | | | |  | 1.410 | 0.235 |
|  | < 1000 | 23 (20.2%) | | 91 (79.8%) |  |  |
|  | ≥ 1000 | 7 (12.7%) | | 48 (87.3%) |  |  |
| Duration of smoking (years) | |  | |  | 21.084 | 0.001** |
|  | < 10 | 13 (38.2) | | 21 (61.8) |  |  |
|  | 10 – 19 | 9 (31) | | 20 (69) |  |  |
|  | 20 – 29 | 1 (5.3) | | 18 (94.7) |  |  |
|  | 30 – 39 | 1(5) | | 19 (95) |  |  |
|  | 40 – 49 | 3(8.8) | | 31 (91.2) |  |  |
|  | >50 | 3 (9.1) | | 30 (90.9) |  |  |

*Fisher’s Exact Test **statistically significant at p<0.05

## Supplementary Content 2: Ethnicity classification

**Caste/Ethnic Groupings**

1. **Dalits**
   - Hill: Kami, Damai, Sarki, Badi
   - Terai: Chamar, Mushar, Dhusah/Paswan, Tatma, Khatway, Bantar, Dom, Chidimar, Dhobi, Halkhor
2. **Disadvantaged janajatis**
   - Hill: Magar, Tamang, Rai, Limbu, Sherpa, Bhote, Walung, Byansi, Hyolomo, Garrti/Bhujel, Kumal, Sinsar, Baramu, Pahari, Yakkah Chhantal, Jirel, Darai, Dura Majhi, Danuwar, Thami, Lepcha, Chepang, Bote, Raji, Hayu, Raute, Kusunda
   - Terai: Tharu, Dhanuk, Rajbansi, Tajpuriya, Gangai, Dhimarl, Mache, Kisan, Munda Santhal/Satar, Dhangad/Jhangad, Koche, Pattarkatta/Kusbadiay
3. **Disadvantaged non-dalit Terai caste groups**
   - Yadav, Teli, Kalwar, Sudhi, Sonar, Lohar, Koiri, Kurmi, Kanu, Haluwai, Hajam/Thakur, Badhe, Rajba, Kewat, Mallah, Nuniya, kumhar, Kahar, Lodhar, Bing/Banda, Bhediyar, Mali, Kumar, Dhunia
4. **Religious minorities**
   - Muslims, Churoute
5. **Relatively advantaged janajatis**
   - Newar, Thakali, Gurung
6. **Upper caste groups**
   - Brahman (Hill), Chhetri, Thakuri, Sanyasi, Brahman (Terai), Rajput, Kayastha, Baniya, Marwadi, Janine, Nuraang, Bengali

## Supplementary Content 3: Informed consent form in English

Namaskar!

My name is **Santosh Shrestha.** I am student of Bachelor in Public Health of Chitwan Medical College, Chitwan. I am conducting my research on **“Perception of graphic health warning and its impact on smoking behavior among residents of Parbat, Nepal”.** I would really appreciate your participation in this research. It will take around 15-20 minutes to fill up this questionnaire. Whatever information you provide will be kept confidential and findings of the study will be used only for the study purpose.

The study procedure involves no foreseeable risk and harm to you. You are allowed to leave the study any time. Your participation in this study is voluntary. However, I hope that you will participate in this study survey since your views are very crucial for this research.

Participation: (a) Participate

(b) Do not participate

## Supplementary Content 4: Interview questionnaire in English

**Section 1: Identification detail**

- 1. Sex of the respondent: 1. Male 2. Female
  2. Age of the respondent…………………. Years completed
  3. Ethnicity:

a. Dalits b. Disadvantaged janjatis c. Disadvantaged non-dalits d. religious minorities e. Relatively advantaged janjatis f. Upper caste groups

- 1. Religion: 1. Hindu 2. Islam 3. Buddhist 4. Christian 5. Others (Specify)…………
  2. Educational status:

a. Illiterate b. Primary school( Grade 1 to 5) c. Middle school (Grade 6 to 8) d. High school (Grade 9 and 10) e. Intermediate or post high-school diploma (Grade 11 and 12) f. Graduate or post graduate g. Profession or honours

- 1. Occupation of respondent

1. Unemployed b) Unskilled worker c) Semi-skilled worker

d) Skilled worker e) Clerical, shop-owner, farmer f) Semi-profession g) profession

- 1. Monthly income of the family (in Rs.)

1. ≤ 2300 b) 2301-6850 c) 6851-11450 d) 11451- 17150 e) 17151- 22850 f) 22851 – 45750 g) ≥ 45751
   1. Marital status: 1. Unmarried 2. Married 3. Divorced 4.Widow/er 5. Separated
   2. Family type 1. Nuclear 2. Joint

1.10 What was your age when you first started smoking? ……………………. Year

1.11 How many years it’s been since you started smoking? …………………. Years

1.12 How many sticks of cigarette you smoke in a day?

1.12 How much you spend on smoking every month? ……………. rupees

**Section 2: Awareness on graphic health warnings**

- 1. Have you noticed graphic health warning on cigarette packages?
  2. Have you heard of any regulation regarding provision to use graphic health warning on cigarette packages?

**Section 3: Perception of graphic health warnings**

3.1 Is graphic health warning labels on cigarette package noticeable?

3.2 Is graphic health warning labels on the package informative?

3.3 Is graphic health warning labels on the package believable?

3.4 Do you feel that it is necessary to keep graphic health warning on package of cigarette?

3.5 Have you ever thought about what the graphic health warnings on the packages have to say?

3.6 What do you think about the effect of keeping graphic health warnings on cigarette packages? (Multiple choice)

3.6.1. It informs about specific health consequences of smoking.

3.6.2 It motivates smokers to quit smoking.

3.6.3 It encourage to reduce no. of sticks smoked per day.

3.6.4 It deters potential smokers from starting to smoke.

3.6.5 It doesn’t have any effect.

3.6.6 any others

3.7 Do you think graphic health warning labels on cigarettes make smokers more likely to quit smoking?

3.8 Have the graphic health warnings increased your awareness on health consequences of smoking?

3.9 Do you think the graphic health warning labels depict the health consequences of smoking?

3.10 Do those health warnings make you think about health consequences of smoking?

3.11 Have you ever talked about the graphic warning labels with other smokers or non-smokers?

3.12 Do you think graphic health warnings make cigarette packages less attractive?

3.13 Do you think graphic health warnings make smoking less attractive?

**Section 4: Change in smoking behavior due to graphic health warnings**

4.1 Do any of the following statements describes your change in smoking behavior since you started to smoke from packages with graphic health warnings? (Multiple choice)

4.1.1 Stub-out cigarette before you smoked completely

4.1.2 Forego a cigarette

4.1.5 Smoke less around other people

4.1.6 Avoid looking at the warning labels

4.1.6 Think about quitting smoking

4.1.7 Feel more vulnerable after seeing the pack

4.1.8 Others………..

4.1.9 No change in smoking behavior

4.2 Have you attempted to quit smoking due to graphic health warnings on cigarette packages?

4.3 Are you planning to quit smoking due to graphic health warning on cigarette packages?

- Within 30 days
- Within 6 months
- Longer than 6 months
- I don’t have any plan to quit

4.5 Have you changed the no. of cigarette sticks smoked per day since you started to smoke from packages with graphic health warnings?

4.6 If you have changed the no. of cigarette smoked per day then,

- Decreased no. of cigarette sticks smoked per day
- Increased no. of cigarette sticks smoked per day

4.7 Will you smoke even if the price of cigarette get doubled?

4.8 Do you feel that taxation on cigarette and tobacco products is essential?
